# MEASURING GENERATIONAL DIFFERENCES IN INTERGENERATIONAL CONTACT WITH A NEWLY VALIDATED SCALE

**DOI:** 10.1093/geroni/igac059.043

**Published:** 2022-12-20

**Authors:** Shannon Jarrott, Shelbie Turner, Karen Hooker

**Affiliations:** The Ohio State University, Columbus, Ohio, United States; Weill Cornell Medical College, Pitman, New Jersey, United States; Oregon State University, Corvallis, Oregon, United States

## Abstract

2. Intergenerational contact influences attitudes about aging, but measures vary in breadth, depth, and psychometrics. We developed and validated the Intergenerational Contact survey of key contact dimensions using online samples. Here, we address: how do young and older adults differ in location and ratings of intergenerational contact? Young (n=433) and older adults (n=286) reported intergenerational contact settings and rated 18 items regarding contact (e.g., “I have something to offer younger/older adults.”) with family and non-family members. Neighborhoods were a common setting for interacting with family and non-family members of different ages. Young adults reported higher negative contact (p<.001) and lower positive contact (p<.001) with related and unrelated older adults than older adults reporting having with younger adults. Developmental theory may partially explain these differences; we address next steps to explore how differences may affect interest in or avoidance of intergenerational contact, associated impacts, and potential interventions.

